# Comparison of Confocal and Super-Resolution Reflectance Imaging of Metal Oxide Nanoparticles

**DOI:** 10.1371/journal.pone.0159980

**Published:** 2016-10-03

**Authors:** Emily J. Guggenheim, Abdullah Khan, Jeremy Pike, Lynne Chang, Iseult Lynch, Joshua Z. Rappoport

**Affiliations:** 1 Physical Science of Imaging in the Biomedical Sciences (PSIBS) Doctoral Training Centre (DTC), Birmingham, Edgbaston, United Kingdom; 2 School of Biosciences, University of Birmingham, Edgbaston, Birmingham, United Kingdom; 3 Nikon Instruments, Inc. Melville, New York, United States of America; 4 School of Geography, Earth and Environmental Sciences, University of Birmingham, Edgbaston, United Kingdom; 5 Center for Advanced Microscopy, and Nikon Imaging Center, Feinberg School of Medicine, Northwestern University, Chicago, Illinois, United States of America; University of California Berkeley, UNITED STATES

## Abstract

The potential for human exposure to manufactured nanoparticles (NPs) has increased in recent years, in part through the incorporation of engineered particles into a wide range of commercial goods and medical applications. NP are ideal candidates for use as therapeutic and diagnostic tools within biomedicine, however concern exists regarding their efficacy and safety. Thus, developing techniques for the investigation of NP uptake into cells is critically important. Current intracellular NP investigations rely on the use of either Transmission Electron Microscopy (TEM), which provides ultrahigh resolution, but involves cumbersome sample preparation rendering the technique incompatible with live cell imaging, or fluorescent labelling, which suffers from photobleaching, poor bioconjugation and, often, alteration of NP surface properties. Reflected light imaging provides an alternative non-destructive label free technique well suited, but not limited to, the visualisation of NP uptake within model systems, such as cells. Confocal reflectance microscopy provides optical sectioning and live imaging capabilities, with little sample preparation. However confocal microscopy is diffraction limited, thus the X-Y resolution is restricted to ~250 nm, substantially larger than the <100 nm size of NPs. Techniques such as super-resolution light microscopy overcome this fundamental limitation, providing increased X-Y resolution. The use of Reflectance SIM (R-SIM) for NP imaging has previously only been demonstrated on custom built microscopes, restricting the widespread use and limiting NP investigations. This paper demonstrates the use of a commercial SIM microscope for the acquisition of super-resolution reflectance data with X-Y resolution of 115 nm, a greater than two-fold increase compared to that attainable with RCM. This increase in resolution is advantageous for visualising small closely spaced structures, such as NP clusters, previously unresolvable by RCM. This is advantageous when investigating the subcellular trafficking of NP within fluorescently labelled cellular compartments. NP signal can be observed using RCM, R-SIM and TEM and a direct comparison is presented. Each of these techniques has its own benefits and limitations; RCM and R-SIM provide novel complementary information while the combination of modalities provides a unique opportunity to gain additional information regarding NP uptake. The use of multiple imaging methods therefore greatly enhances the range of NPs that can be studied under label-free conditions.

## Introduction

Naturally occurring nanoparticles (NPs) have always existed in the environment, derived from sources including volcanic dust, soil and sediment [1]. However, recent years have seen an increase in the incorporation of man-made NPs into commercial goods, including sun screen, chewing gum, tennis rackets, and wrinkle-free shirts, in addition to their use as additives in industrial processes, such as cerium dioxide NPs or nanoceria use in diesel fuel [2–5]. The use of NPs within biomedicine for diagnostics and intracellular delivery of therapeutics is a key application of nanotechnology. NPs offer unique properties such as large surface area to mass ratio, quantum properties and ability to adsorb, carry and release therapeutic payloads. Several drugs have been combined with NPs, particularly for cancer therapy, including paclitaxel, doxorubicin and methotrexate [6–8]. These NPs can be targeted, passively and actively, to specific sites of interest, decreasing off-target toxicity associated with conventional chemotherapy. In addition, super-paramagnetic iron oxide NPs (SPIONs) can be used as Magnetic Resonance Imaging contrast agents and for magnetically guided drug delivery and release [9–11]. Despite the advantages they offer, a large majority of NPs fail to increase drug efficacy in the clinic [12]. A crucial factor limiting the success of these NP is the inadequate understanding of NP-cell interactions, and little is known about the short and long term health effects of NP exposure [13]. Therefore there is an increasing drive for the development of research methods with throughput sufficient to facilitate safe use of these NPs and to deliver their full potential as diagnostics and therapeutic agents. Development of research methods that can be applied to further the understanding of NP-cell interactions, including potential mechanisms of NP entry and toxicity, is therefore a critical research priority.

TEM remains the current gold standard for NP imaging. Electrodense NPs, such as gold, have often been used to increase contrast within TEM micrographs, such as in immunogold labelling of proteins of interest [14,15]. TEM provides ultrahigh resolution capable of distinguishing individual mono-disperse intracellular NPs, and therefore provides a means of quantifying NP number; this quantification has been applied to multiple NPs including SPIONs, silica, zinc and gold [16–20]. Despite the advantages that TEM offers, conventional sample preparation is extensive and leads to the alteration of cellular structure and morphology during the dehydration and resin embedding processes, and limits investigation to fixed samples. TEM is therefore limited in its capacity to provide the high throughput studies necessary for the investigation of NPs cellular interactions, and data interpretation is complicated due to poor contrast arising from soft materials [21].

Alternatively, fluorescent NP labelling and subsequent imaging can be used for investigation of NP internalisation and location of intracellular NPs. This method requires little sample preparation; however the use of fluorescent tags has known limitations, including low sensitivity, difficult bioconjugation, low quantum efficiency and photobleaching effects, in addition to potential alteration of NP surface chemistry if the label is surface-attached, a factor known to influence biological activity [22–24]. Fluorescent tags can also dissociate from the NP following uptake, complicating the experimental analysis and results [25]. An alternative technique that can be used in conjunction with fluorescent imaging is reflected light imaging, in which metallic NP give rise to significant contrast [26]. Thus, the use of reflectance imaging obviates the need for fluorescent NP labelling, and provides an alternative platform for visualising these NPs [26]. Reflectance imaging can be performed with a variety of modalities including widefield illumination, Confocal Laser Scanning Microscopy (CLSM), Total Internal Reflectance Microscopy (TIRM), Structured Illumination Microscopy (SIM) and Optical Coherence Tomography [27–31]. CLSM uses pinhole optics to block out-of-focus signal from reaching the detector, effectively de-blurring images, while Reflectance Confocal Microscopy (RCM) provides a simple contrast enhancement method for interrogation of a variety of unlabelled samples, including those containing metallic NPs [31–36].

RCM provides a quick acquisition with little sample preparation and is therefore applicable to both fixed and live cells. The key function of CLSM and RCM is the optical sectioning capability, providing increased resolution, compared to epifluorescence, with depth selectivity. The optical section thickness depends upon the excitation laser wavelength, numerical aperture (NA) and the refractive index of the sample medium. This allows semi-thick samples to be imaged throughout, providing a 3D volume of the area of interest. A key benefit of CLSM reflectance imaging is the potential correlation with Fluorescence Confocal Microscopy (FCM) data [36]. The use of the same sample preparation and acquisition instrumentation allows further information to be obtained than with either modality on its own. This provides an opportunity to visualise NP uptake using reflectance, in conjunction with fluorescent labelling to assess the compartment localisation or cell perturbations resulting from exposure, such as alteration of cell viability [37–39]. Fluorescent compartment labelling provides an important technique often used to elucidate cellular trafficking mechanisms of NPs; determining these routes of uptake is critical in ensuring the subsequent success of NPs in personalised therapies [37]. Despite the advantages CLSM methodologies provide, the resolution is fundamentally constrained due to the finite wavelength of the incident light, and therefore NPs and NP clusters appear as blurred spots [40–42]. Light microscopes, such as RCM can resolve objects laterally that are separated by roughly 250 nm, and axially by approximately 600 nm. This, however, relies on perfect experimental conditions and the concept of an infinitely small pinhole, and therefore will not be achieved in practice, particularly in fluorescence microscopy where light intensity is a limiting factor [43]. Therefore, structures separated by less than the resolution limit of the system will not be resolved. To overcome this limitation, novel methods are necessary for accurate NP studies.

There are methods available that can be used to increase the resolution of light microscopy, collectively referred to as “super-resolution microscopy”, that bridge the gap between light and electron microscopies [44–46]. One method, first proposed by Neil *et al*, called Structured Illumination Microscopy (SIM), uses grid projections to effectively increase the resolution limit imposed by diffraction [44]. In a conventional SIM acquisition, a grid pattern is projected onto the sample in several different orientations, and the emitted fluorescence signal is detected and reconstructed in the Fourier domain into the final image [44]. SIM can increase resolution two-fold or more, enabling the identification of previously unresolvable cellular structures such as individual nuclear pore complexes [47,48]. SIM can therefore provide the necessary advantages / resolution for imaging NP-cell interactions; however, while super-resolution reflectance techniques have been described they are currently limited to custom built microscopes, limiting widespread use [27].

The increasing use of NPs in a range of consumer and medical applications has driven the need for an in-depth understanding of their biological behaviours and effects following cellular exposure, for both safe and efficacious use. Reflectance microscopy provides a unique opportunity for the investigation of these particles and, in conjunction with fluorescence compartment labelling, can provide insights into their cellular entry, trafficking, fate and toxicity [37,38,49]. Optimisation of the imaging procedures for reflectance methods is described herein, and a workflow for the visualisation of NP uptake, via the sequential acquisition of RCM images and super-resolution R-SIM images followed by processing and TEM imaging, is described. Despite the advantages offered by RCM, such as fast acquisition of large sample areas with optical sectioning capabilities, the resolution is not as high as super-resolution techniques. This paper presents the first utilisation of a commercial N-SIM module to acquire reflectance imaging data with greater than 2-fold increase in resolution compared to RCM, applied to the investigation of NP uptake and cellular localisation. This allows clusters of NP separated by ~115 nm to be resolved. This is a necessity for accurate internalisation and colocalisation studies to determine the precise uptake, localisation and trafficking route of NPs. Within this study, cancer cell models are employed, which are an important model system for understanding cancer diagnostics and drug delivery. The potential for quantification of uptake, visualization of trafficking and the ultimate destination of particles, as well as for assessment of potential removal and/or degradation/dissolution of the NPs will be instrumental in the development of therapeutic NP agents. R-SIM as a method is validated by the comparison to RCM and TEM, confirming cellular entry of electrodense NPs and their subcellular localisation and fate. Together, the combination of imaging techniques maximises the information gained from a sample regarding NP uptake route, uptake form (single NPs or NP clusters) and sub-cellular localisation.

The studies presented here have made use of two different metallic NPs of significant biomedical and environmental interest, SPIONs and cerium dioxide NPs, and we have been able to visualize both NPs through all three means, revealing information about the cellular uptake of these NPs as well as insights into the relative merits of these different imaging techniques.

## Results and Discussion

### Transmission Electron Microscopy

**Fig 1:**
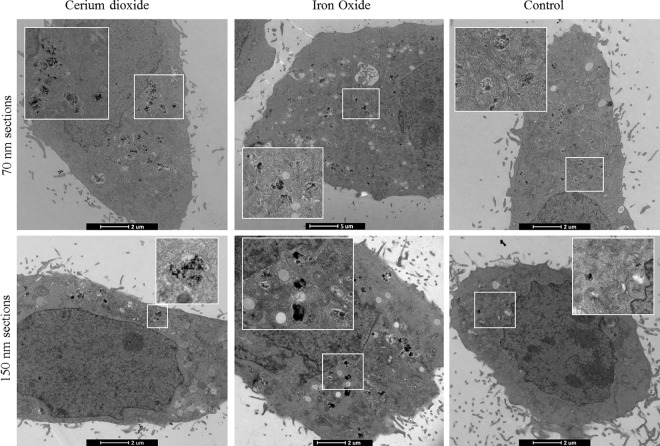
**Electron micrographs of A) cerium dioxide NPs, B) SPIONs, and C) Non-treated cells.** The top panel depicts 70 nm ultrathin sections, the standard TEM mode, while the bottom panel uses 150 nm sections. Ultrathin and semi-thick sections can thus be successfully imaged. Thin sections give rise to a crisper image, with increased contrast visible at organelle boundaries. Thick sections have slightly less contrast due to the denser area being imaged but allow alignment with confocal slices (see below).

Fig 1 shows imaging of intracellular NPs using TEM. Traditionally, ultrathin (70 nm sections) are imaged using TEM, generally to achieve the highest possible definition and contrast at the boundaries of organelles present in the cell cytoplasm (Fig 1 top panel). Thick sections (150 nm) can also be obtained and imaged with high power TEMs (Fig 1 bottom panel). This leads to acquisition of a TEM slice with increased density of particles in a particular location, but additionally increased density of cellular constituents. Electrodense NPs, such as iron oxide and cerium dioxide give rise to contrast within the electron micrograph, alone or inside cells on both thick and thin sections (Fig 1 and S1 Fig).

### Reflectance Microscopy

**Fig 2:**
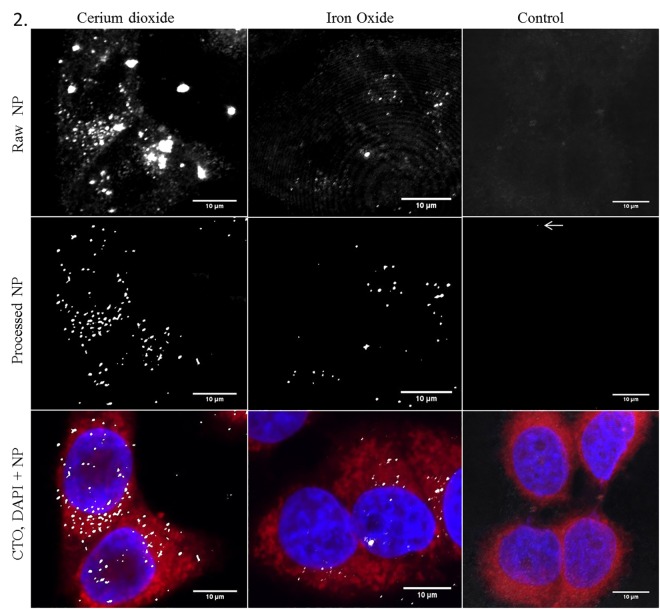
RCM allows visualisation of NP uptake within cancer cell models. NP uptake can be visualised in HeLa cells treated with cerium dioxide NPs (65 cells) or SPIONs (51 cells) comparted to untreated control cells (25 cells). Images show maximum intensity Z-projections of cells stained with Cell Tracker Deep Red (CTDR) (red), 4’,6-diamdino-2phenylindeo (DAPI) nuclear stain (blue) and NP reflectance signal (grey). Control cells show no high intensity reflective spots. The raw intensity reflectance images show background reflectance in both control and treated cells (top panel). Following post processing, regions of high intensity signal are segmented from background signal (middle panel). Overlay of fluorescence stains and segmented reflectance NP signal (grey) (bottom panel).

The innately different optical densities or refractive indices (RI) within non-homogeneous samples introduce contrast into the acquired RCM image [50,51]. Fig 2 shows the CLSM reflectance and fluorescence images of SPION and cerium dioxide NPs when utilising a 100X objective with Nyquist sampling rates to achieve maximal resolution. Both NPs provide a substantial RI difference compared to the surrounding media and therefore provide excellent contrast, allowing detection in cancer cell models (Fig 2). When comparing imaging media, we found Vectashield mounting medium (RI 1.44) rather than hard set media (Prolong Gold) or aqueous buffers such as Phosphate Buffered Saline (PBS) (RI 1.33) improved the signal-to-noise ratio (SNR) in fixed samples. When utilising lower magnification objectives RCM can allow acquisition of large sample areas which permits multiple cells to be imaged simultaneously. This can be done using large image tiling to prevent loss of resolution (0.5mm^2^ in 40 minutes). Therefore, RCM provides a unique opportunity to investigate the cellular effects of NP uptake in a time efficient manner critical for high throughput investigation. Fig 2 shows representative results of the processed RCM images, following the use of Gaussian smoothing and intensity clustering and segmentation, providing assessment of uptake of NPs relative to untreated control cells (S2A Fig). Although there appears no observable NP signal in the control cells automated algorithms, such as those demonstrated here, can detect regions of contrast arising from cell constituents otherwise undetectable by eye (Fig 2 and S2 Fig, white arrow). This is evidenced by the slightly higher than ‘zero’ regions of intensity detected in control cells when a sample of cells are analysed. It is therefore crucial that untreated cells (Fig 2) are employed as a baseline negative control in order to compare NP-based signal relative to inherent cellular reflection (Fig 2 and S2 Fig).

### Reflectance structured illumination microscopy

**Fig 3:**
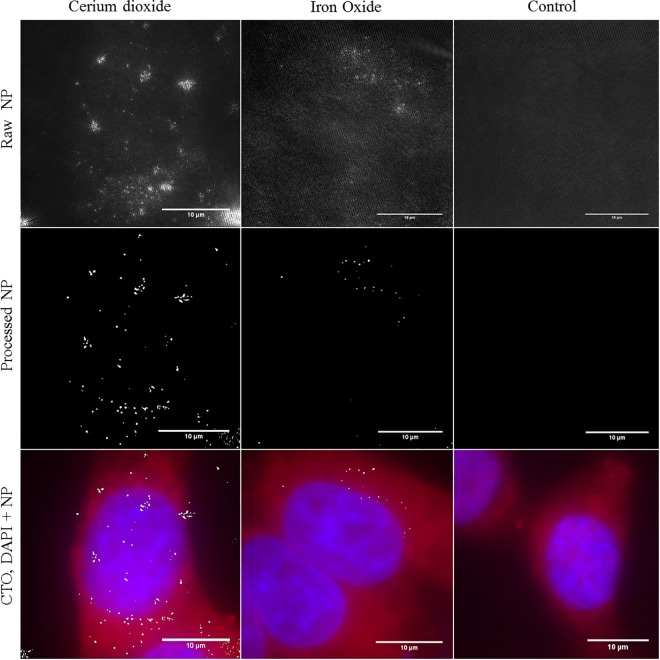
R-SIM allows visualisation of NP uptake at increased resolution. Reflectant signal was visualised in HeLa cells treated with cerium dioxide NPs (86 cells), SPIONs (32 cells) or untreated (25 cells). Images show maximum intensity Z-projections. Fluorescence images show conventional widefield epi-fluorescence of cells stained with CTDR (red) and DAPI nuclear stain (blue). Reflectance images show 2D SIM acquisition of reflectance NP signal (grey). Control cells show no high intensity reflective spots. The raw intensity reflectance images (top panel) show background reflectance in control and treated cells. Post processing and segmentation can isolate regions of high intensity reflectance (NPs) from background signal (middle panel). Overlay of cells stained with CTDR (red), DAPI nuclear stain (blue), and segmented reflectance NP signal (grey) (bottom panel).

SIM is an established super-resolution microscopy technique that provides more than two-fold increased resolution relative to conventional light microscopes [44,46]. A typical SIM acquisition consists of 9 or 15 (2D or 3D) acquisitions at different grid angles and phases. These images can be processed in reciprocal space to reconstruct the Fourier transform (FT) image with high resolution information, the inverse of which is the super-resolution image. The theory of SIM is described in detail in references [44,46]. Reflectance imaging of NPs has previously been demonstrated using a custom made SIM [27]. We have found that the collection of reflected light can be achieved on the commercially available Nikon N-SIM system (Fig 3).

In a typical fluorescence SIM acquisition, the filter cube placed within the light path consists of an excitation filter, emission or barrier filter and a dichroic mirror. This filter cube separates the fluorescent light emitted from the specimen for detection, from the light used to excite the specimen. This enables detection of emitted fluorescent light from the sample. However, for reflectance imaging this type of spectral separation is not employed. Instead, we utilized a filter cube that contains a half-mirror in place of the dichroic, which permits passage of light that is reflected back from the sample to the detector, rather than selectively allowing passage of a particular wavelength of emitted light. This provides the first report of a commercial SIM microscope being used for super-resolution imaging of intracellular reflectant (NP) structures, utilising the half-mirror filter cube. This is advantageous particularly for the imaging of non-fluorescent NP, such as SPIONs and cerium dioxide NPs, which would otherwise be undetectable using fluorescent SIM, and therefore negates the need for fluorescent modification and labelling of the NPs. R-SIM has been successfully used to image four different NPs including two types of SPIONs, polystyrene and cerium dioxide NPs (data not shown), indicating the breadth of application for this technique. However, despite the advantages SIM offers for label free imaging, it currently lacks the ability to differentiate between different types of relfectant objects.

When optimizing parameters for R-SIM imaging, we obtained our best SNR with 488 nm incident light and use of Vectashield mounting media relative to alternative approaches such as aqueous imaging media. Thus, R-SIM can be performed with the same sample preparation conditions as RCM. However, like RCM, there will still be small amounts of detectable signal arising from the inherent cellular reflection, therefore untreated control cells must be employed to visualise baseline reflectance to draw accurate comparisons and conclusions. Fig 3 shows the raw and processed R-SIM images of intracellular SPIONs, cerium dioxide NPs and untreated control cells. Segmentation was achieved using intensity based clustering to segment signal and assess the detection and uptake of NPs relative to control cells (S2A Fig).

In order to make qualitative conclusions regarding the advantages offered by R-SIM, cells were imaged sequentially with RCM, and then R-SIM following incubation with NPs as detailed in the methods section; and subsequently processed for TEM. S1 and S2 Boxes provide a summary of the experimental conclusions determined for RCM and R-SIM acquisitions; Table 1 provides numerical values regarding the various acquisition specifications, indicating the pros and cons of each method.

**Table 1:** Comparison of the image acquisition specifications for each modality. Table 1.provides a comparison of the image acquisition specifications for each modality.

	Max observed X-Y Resolution	Max observed Z–Resolution	Theoretical Optical thickness	Live	Large Image	3D volume
RCM	>340 nm	1077 nm	~480 nm	Yes	Yes (~ 1 hour)	Z-Stacks
R-SIM	>115 nm	685 nm	~685 nm*	Yes	Yes (~24 hours)	Z-Stacks
TEM	>4 nm	NA	~70–150 nm	No	No	Serial Sections / Tomography

*Approximated by the Z-PFS as this greatly depends on the RI of the sample and the extent of out of focus light.

**Fig 4:**
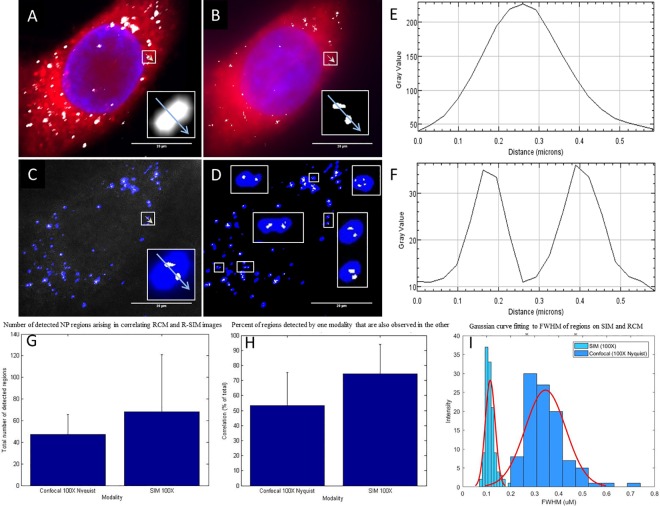
Correlation of data obtained from RCM and SIM reflectance. Maximum intensity Z-projection images of a HeLa cells treated with cerium dioxide NPs, acquired with RCM and R-SIM using identical 100X, 1.49 NA objective. RCM imaging volume is 3.6 μm and SIM 4 μm. Images A) (RCM) and B) (R-SIM) show CTDR (red) cytoplasmic stain, DAPI (blue) nuclear stain and NP signal (grey). Overlay of the cerium dioxide NP regions show particles detected in RCM (blue) and SIM (grey) in both the raw (C) and processed (D) images. White boxes display a sample of regions where RCM detects one spot and SIM detects multiple spots, illustrating the enhanced resolution of SIM. Intensity line scans of RCM (E) and R-SIM (F) show the decrease in peak width in SIM and the detection of two peaks where RCM detects one. The average total number of regions detected via each technique was computed (47 and 68 for RCM and R-SIM respectively) (G). The percentage of ‘regions’ or ‘connected components’ visualised with each modality, RCM and R-SIM, that are also seen in the other modality (54% and 74% respectively), were computed automatically using MATLAB as detailed in the methods section using 27 cells from multiple experiments performed on separate days (H). The means and STD are plotted. Comparison of the size distribution of the FWHM of 100 / 125 regions for RCM and R-SIM respectively are shown (I), with a fitted probability density function.

The dramatically increased resolving power of the R-SIM technique is evidenced by the appearance of multiple smaller structures on the R-SIM image, where RCM shows single correspondingly larger structures at the same locations when using both the 60X (S3 Fig) and the 100X objectives for RCM (Fig 4C and 4D). Thus, the higher resolution allows separation of regions that would otherwise be non-resolvable using RCM alone. This is also evidenced in the line intensity profiles (Fig 4E and 4F) of the selected region depicted on the raw intensity images (blue arrow in white box; depicted in Fig 4A and 4C). The RCM line-scan shows a single broad peak (Fig 4E), where the R-SIM line scan displays two peaks at the same location (Fig 4F). The decrease in the width of the detected R-SIM peaks indicates a decrease in the FWHM, which is often used as a measure of resolution. The FWHM for regions detected by RCM using the 100X 1.49 NA objective with Nyquist sampling is 345 nm +/- 83 nm averaged over 100 regions. The average FWHM for regions detected by SIM reflectance is 115 nm +/- 21 nm averaged over 125 regions. This is substantially better than the theoretical maximal X-Y resolution offered by confocal systems: ~250 nm.

When comparing images obtained with RCM using the 100X objective to those taken with RCM using the 60X, the 100X performs substantially better; regions detected using the 100X objective with RCM align to those detected by R-SIM with little to no offset, indicating the power of utilising the same objective (Fig 4 and S3 Fig). R-SIM did not appear to reveal as many isolated regions as RCM. This is evident in the image shown where the majority of regions detected with R-SIM correspond to a region detected by RCM (Fig 4). This can be quantified as a percentage of total ‘objects’ detected. Pixel based methods are not appropriate due to the difference in resolution. Objects that are detected within R-SIM images are also observed with RCM with an average of 74% across 27 analysed cells (Fig 4H). Conversely, 54% of regions detected on RCM correlate to regions detected with R-SIM (Fig 4H). Although volumes of approximately the same regions were acquired with corresponding Z-step size, the identical volumes cannot be directly compared, likely leading to some of the discrepancies seen. The perceived centre of the detected region is not perfectly aligned in all cases; this may, in part, relate to the different reflected rays that are collected from irregular shaped NP and agglomerates under the different illumination patterns. Agglomerate shape, size and orientation with respect to the optical axis are all known factors that contribute to the angle of reflectance, and therefore the perceived centre of detection [52–54]. Additionally, problems may arise from the dish not being completely flat during acquisitions. Thus although SIM provides better resolution, RCM permits identification of more NP positive structures.

The total signal detected when imaging cerium dioxide NPs is increased with R-SIM when compared to RCM (S2 Fig). This is expected if a resolution increase is observed, due to the resolving of smaller structures previously detected as one with RCM. Interestingly, the detection of SPIONs remains roughly the same. It is likely that this discrepancy results from the low intensity reflectance signal generation associated with these particular SPIONs. The core size of the SPIONs used in this study is small (4 nm) and irregularly shaped, as seen on the TEM micrograph (S1 Fig). These results indicate that particle material, size and homogeneity, in part, determine their detection capacity with R-SIM. Therefore it would be of interest to synthesise NPs of varying material, size, shape and homogeneity and subsequently systematically compare the detection of these with RCM and R-SIM. This could be used to infer conclusions regarding the limitations of R-SIM in terms of NP physical properties. The difference in detection can somewhat be explained by the different scattered light collection methods used in each optical sectioning technique. In RCM, out-of-focus light is filtered by the conjugate pinhole system, resulting in an increase in image quality, greater depth of field and increased particle detection. SIM, however, utilises widefield illumination at multiple angles and phases, and therefore suffers from the detection of out–of-focus blur; only high contrast in focus signal will be successfully reconstructed, leading to a narrower plane of particle inclusion [44]. The reconstruction algorithm used for performing the reconstruction is part of proprietary Nikon software designed primarily for fluorescence imaging, which cannot readily be replaced or modified by the user. Reflected light images can include high levels of background scattering from cellular components, which the reconstruction software is not designed to deal with. Thus, it is likely that this background scatter leads to obscuring of signal, especially in the case of the smaller, irregularly shaped SPIONs. This can limit particle identification. Potential future changes to the image processing and reconstruction algorithm may lead to increased image quality and increased detection. Despite this limitation, these analyses indicate the increased detection power of RCM, relative to the significantly improved resolution of SIM, suggesting that their combined application on a single sample will provide maximal information.

**Fig 5:**
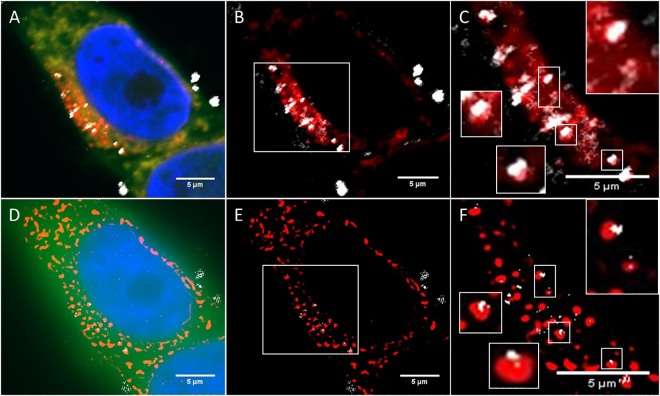
Colocalisation of NP signal with the lysosome following 24 hour NP incubation. Single optical slice images of a HeLa cell treated with cerium dioxide NPs, acquired with RCM (A:C) and R-SIM (D:F). The theoretical slice thickness for RCM is ~480 nm. Theoretical optical slices for R-SIM are approximately the theoretical FWHM ~685 nm. Images A) (RCM) and B) (R-SIM) show CTDR (green) cytoplasmic stain, DAPI (blue) nuclear stain, LysoTracker Red DND-99 lyosomal stain (red) and NP signal (grey). Increased zoom of RCM (B:C) and R-SIM (E:F) show colocalisation of NP signal with fluorescent lysosomal signal (red). It is difficult to discern colocalisation with the RCM image; however the use of SIM provides proof of colocalisation in some cases (white boxes).

To further demonstrate the applicability of R-SIM, we applied the technique to the study the intracellular trafficking of NPs within HeLa cancer cells. Understanding of the uptake and trafficking of NPs is crucial to their future success in biomedicine as drug delivery agents and as successful diagnostic tools. NPs are typically internalized in vesicles and transported into the cell through a variety of endocytic pathways. It had previously been demonstrated that the size of the NP itself can influence the pathway employed for internalisation [55]. Clathrin mediated and macropinocytosis pathways are known to accumulate internalised NP in compartments with low pH (such as the lysosome), a property that may be exploited in the drug delivery process for pH sensitive drug release [56]. When used in conjunction with fluorescence staining, reflectance imaging can be used to determine the localisation of NP within various compartments. These colocalisation investigations are often used to investigate particle internalisation routes, however the sensitivity and accuracy is limited by the resolution of the imaging modality. As demonstrated in S4 Fig and S1 Video, trafficking of cerium dioxide NPs into lysosomal compartments can be visualized in live cells in real time using RCM. The NPs under investigation within this workflow have reflectant core sizes of 4 and 6 nm respectively, this is significantly smaller than the maximal resolution available with RCM and individual NPs will not be resolved within NP clusters. RCM can only realistically distinguish between clusters of NP and labelled compartments that are separated by ~340 nm or more in the X-Y and 800 nm in the Z-direction [40–42]. R-SIM can provide increased accuracy for colocalisation studies, due to the greater than two-fold resolution increase relative to RCM. Fig 5 shows RCM (Fig 5A–5C) and SIM (Fig 5D–5F) images of NP uptake (grey) in cells with labelled lysosomes (red). Colocalisation of SPIONs within the lysosome can be seen in both cases. R-SIM provides increased resolution and therefore increased assurance that the signal seen is true colocalisation (Fig 5C and 5F white box). However some regions in the image that appear colocalised with RCM do not actually appear colocalised when imaged using R-SIM, and are likely to be contained within other membrane bound structures (such as endosomes). This technique can be applied to fluorescent labelling of various compartments, to elucidate the NP trafficking and fate within target cells; information that can be used to aid the subsequent design of therapeutic NPs [37]. Despite the increase in resolution offered by R-SIM, individual NPs within clusters will still not be distinguished, due to the small core size of the NP used in this study.

Both reflectance techniques offer different advantages and disadvantages for NP imaging; RCM provides a fast acquisition of large sample regions, allowing high throughput investigations. R-SIM provides the increased resolution necessary for accurate cell trafficking studies, approaching the resolution of single NPs although for ultrasmall particles such as used here there is room for further improvement. TEM remains uniquely able to visualise individual particles and clusters along with other intracellular organelles (Fig 1 and S1 Fig). TEM imaging can therefore be performed following reflectance imaging to not only confirm the uptake of NPs within cells, but also to confirm that reflectance imaging does not damage the cell ultrastructure during the intensive imaging procedures.

### Transmission Electron Microscopy

**Fig 6:**
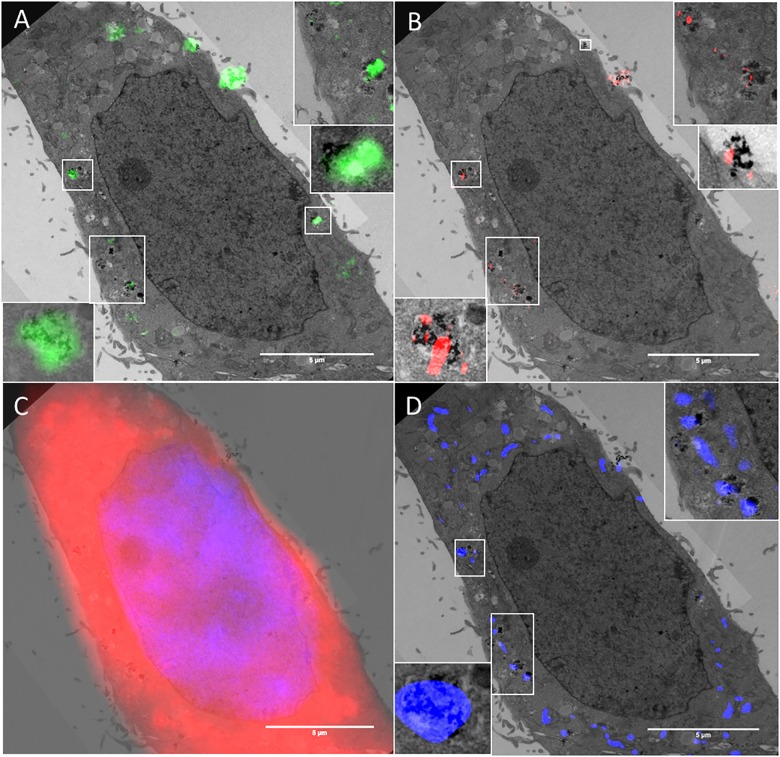
Cellular uptake and localisation of cerium dioxide NPs visualized by RCM, FCM, SIM and TEM. Reflectance and TEM overlays of HeLa cells treated with cerium dioxide NPs. The ultrastructure of the cell is preserved and the sub-cellular localisation of NPs is evidenced by the lysosomal fluorescence stain (D). The TEM image has a section thickness of 150 nm. RCM overlay has a theoretical optical thickness of ~480 nm. R-SIM has an optical thickness of approximately the measured FWHM_axial_ which is 685 nm. Adjacent image sections were combined so that the thickness across modalities was as consistent as possible. Reflectance intensity arising in both RCM (A: Green) and SIM (B: Red) corresponds to regions detected by TEM. Overlays of DAPI nuclear and CTDR cytoplasmic stain (C) are shown. LysoTracker DND-99 stain (D: Blue) shows the localisation of detected NPs in lysosomes. White boxes show regions of correlation between all three modalities with increased magnification.

**Fig 7:**
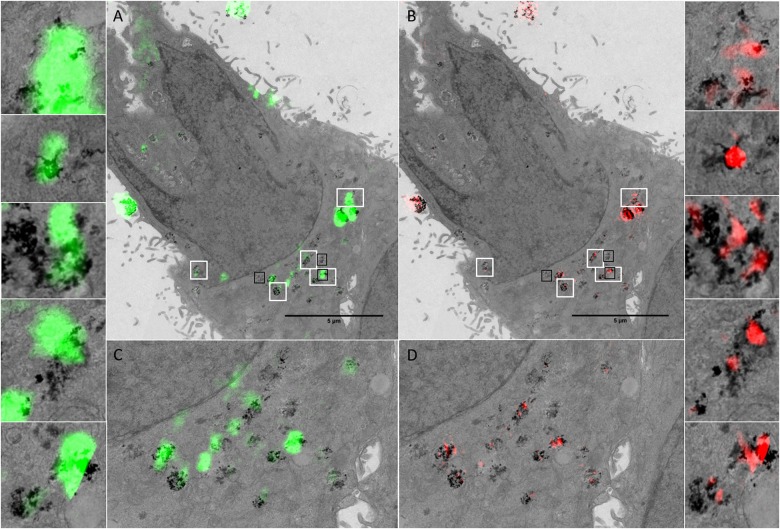
Cellular uptake and localisation of cerium dioxide NPs (Sigma-Aldrich) visualized by RCM, SIM and TEM. Reflectance and TEM overlays of HeLa cells treated with cerium dioxide NPs. The cell outline in both TEM and reflectance microscopy is highly preserved, facilitating identification of the same cell. The ultrastructure of the cell is preserved and the sub-cellular vesicular localisation of NPs is evident. Individual NPs can be visualised at high magnification with TEM. RCM has a theoretical optical thickness of 480 nm. R-SIM has optical thickness approximated by the FWHM_axial_ calculated to be 685 nm. Adjacent image sections were combined so that the thickness across modalities was as consistent as possible. Reflectance intensity arising in both RCM (green; A and C) and SIM (red; B and D) correspond to regions detected by TEM. White boxes show regions of correlation between all three modalities with increased magnification. Black boxes show regions that are presented in S6 Fig.

The TEM image allows visualisation of NPs and NP clusters localised within the cell, thus confirming that NP uptake has indeed occurred. TEM also provides information regarding subcellular localisation, and identity (composition) of the detected reflectant signal if coupled to EDX [57]. TEM images of HeLa cancer cells treated with cerium dioxide NPs were acquired following reflectance imaging allowing visualisation of the preserved ultrastructural detail within the cell and intracellular NPs. In some cases, the intracellular localisation of NPs inside vesicular structures can be seen, providing additional information regarding the subcellular trafficking and localisation of these NPs within the endocytic transport system (Figs 6 and 7 and S7 Fig).

In order to obtain the most accurate comparison possible it is important that the areas being compared are roughly equivalent. When serial sections are imaged, adjacent sections can be registered and merged using a minimum projection; the TEM image in Fig 7 is thus a min-projection of thick serial TEM sections (total thickness 300 nm) (S5 Fig) to create a single image. This TEM ‘composite image’ can then be co-aligned with RCM and SIM optical slices that contain equivalent data. The RCM overlays consist of one optical section of a confocal stack, with a theoretical optical thickness of 480 nm (Figs 6 and 7). The observed FWHM in the Z direction is 1077 nm (S8 Fig). The optical slice for SIM is approximated by the measured Z-PSF, and is larger than the theoretical optical slice thickness of RCM. However, the SIM reconstruction algorithm will only successfully reconstruct high contrast (in-focus) signal near the centre of the plane, leading to a Z-FWHM that is smaller than the Z-FWHM of RCM (685 nm and 1077 nm respectively) when measured over 20 regions (S8 Fig). The effect this has on NP inclusion within the imaged plane was concluded by comparing the number of consecutive planes that particles are present within on matching R-SIM and RCM images. Particles appeared for an average of 7 consecutive optical slices with 0.2 micron increments in RCM, and an average of 4 consecutive optical slices with 0.2 micron increments in R-SIM. For this reason it is beneficial to include multiple optical sections from SIM to ensure that the information is consistent with that provided by one optical slice from RCM. Following alignment, correlation between reflectance signal, both RCM and R-SIM, and TEM signal can be visualised in HeLa cancer cells treated with cerium dioxide NPs (Figs 6 and 7 and S7 Fig).

The signal detected with R-SIM appears to correlate with increased accuracy, in part due to the increase in resolution that allows the separation of nearby clusters (Figs 6 and 7). The signal detected across all three modalities co-occurs with signal from fluorescent lysosomal stain, indicating the localisation of the NPs within the cell (Fig 6). In some cases reflectance signal does not co-occur with signal on the TEM image, this likely corresponds to TEM signal from neighbouring sections; despite the increase in section thickness used in this study the volume is not exactly the same and particles from up to 7 ‘optical slices’ may be included into the 1 RCM slice. Therefore, for further quantifiable correlative studies imaging of multiple serial sections in TEM would be beneficial to create an imaged volume. It would be of interest, in such an experiment, to utilise 3D Correlative Reflectance Electron Microscopy (CREM) to systematically evaluate the number of NPs per reflectance spot, in order to evaluate the sensitivity of each technique.

At magnifications such as 1900X it is difficult to distinguish between NP regions and electrodense cellular matter. When looking at images with higher magnification it becomes more apparent that some of the regions not detected by reflectance methods do not actually contain NPs (S6 Fig). In other cases, small NP clusters may be masked by larger nearby clusters. The light scattered by an object within a sample is dependent upon physical properties such as size and density [53,54]. It is therefore possible that regions not detected on SIM or RCM, but detected on TEM are not sufficiently agglomerated or aggregated and therefore do not give rise to a detectable signal in the reflectance techniques. Additionally, the presence of cellular structures with increased inherent reflectance above or below NPs may effectively mask the signal, in particular with SIM imaging. This effect could be examined further by comparing reflectance and TEM images of cells treated with NPs coated in a dispersing surfactant, thereby decreasing agglomeration, in order to identify a potential decrease in correlation between the reflectance techniques and TEM. It would be important to determine the extent of this masking effect, if it is indeed occurring, as it could prove to be a limitation of this technique for translation to other samples, such as tissue.

Matching image planes were co-aligned using multimodal affine registrations in MATLAB. Fully automated alignment is hindered by alterations in cellular morphology, such as shrinkage, that arise due to the sample preparation methods involved in TEM processing [58,59]. This imposes constraints on the correlation between reflectance image data with high resolution TEM data; cells with irregular shapes or large sizes have areas that may experience shrinkage at different rates compared to the bulk of the cell. In some cases this can limit the realignment of samples. Further post-processing methods can be explored to fully automate the procedure. Semi-automated methods, such as user input of defined points on the images, such as on the cell cytoplasm or nuclear regions, can facilitate increased accuracy for realignment, in addition to simplified processes using MATLAB built in functions, such as CPSelect and imwarp, or ImageJ plug-ins such as TurboReg. Fully automated methods could also be explored, using segmentation and registration of DAPI nuclear regions to decrease alignment time and lead to more accurate conclusions. Coherent Point Drift (CPD) allows registration of point clouds and when applied to cellular features, may facilitate automated alignment of intracellular regions with compensation for shrinkage, however this is currently limited to images that contain distinct features (such as multi nuclear cells) (S9 Fig) [60].

## Conclusions

Metal oxide NPs have wide scale applications from investigative research and the inexpensive *in vivo* detection and screening of diseases such as cancer (e.g. SPIONs) to use in vehicle fuel efficiency (e.g. cerium dioxide NPs) [8,26,61]. Understanding the cellular uptake and potential for toxicity of NPs is critical to ensure successful, effective and safe applications. Reflectance imaging provides a label free method for visualising NP uptake, and metal oxide NPs, such as cerium dioxide and SPIONs, introduce significant contrast into reflected light images, providing opportunity to elucidate the cellular interactions that occur during NP uptake [39]. Reflectance imaging is a time effective investigative method that requires little sample preparation and can readily be used in conjunction with fluorescence imaging. RCM allows acquisition of large amounts of 3D data with apparent increased detection sensitivity when compared to R-SIM. RCM can also be performed with live cells. R-SIM, however, provides a substantial increase in resolving capabilities compared to RCM and distinguishes between NP clusters with an average FWHM of 115 nm compared to 354 nm with RCM. This distinguishes clusters previously unresolvable by other reflected light techniques, such as RCM, affording more accurate investigations and conclusions regarding the presence and location of NP clusters within cells, and permits the superresolution imaging of unlabelled NP that would not be possible with current fluorescent SIM acquisitions. The relatively long effective exposure time of SIM however, ~3 seconds, makes it less compatible with living samples, however it is possible. Together, the combination of RCM and R-SIM techniques can highlight important information regarding the cellular uptake and localization of NPs, with no additional sample preparation needed to transition between the two modalities.

Compared to reflectance imaging, TEM provides superior NP detection capabilities and remains uniquely able to resolve NPs and NP clusters. However, resolution and absolute quantification of individual NPs inside cells requires ultrahigh magnification with TEM and data interpretation is complicated with some materials due to low contrast [21]. TEM involves technically demanding sample preparation steps and long acquisition times, rendering it unsuitable for live or high throughput investigative studies. Thus, the use of reflectance methods provides a cheaper, faster, alternative technique, with detection rates approaching that attained with the gold standard method of TEM. Reflectance imaging is well suited to both fixed and live cell imaging and is capable of optical sectioning through samples and super-resolution investigation (R-SIM). The combination of reflectance image acquisition and subsequent correlation to ultrahigh resolution TEM imaging can increase the confidence that the signal detected by each modality is originating from NP internalisation within cells, and not an artefact from processing and imaging.

The automated analysis we have employed within this study offers significant benefit for NP investigations. The analysis consists of two stages: 1) the automated cell-segmentation facilitated by CellTracker Orange or CellTracker Deep Red, through a process of denoising (Gaussian smoothing) and intensity clustering (K-means) and 2) NP segmentation, consisting of denoising (Gaussian smoothing), background subtraction and intensity clustering. The properties (such as detected spot area, detected spot size, number of spots present and cell intensity) of the resultant segmented cell regions can then be collated to give a thorough picture of NP behaviour following cellular internalisation. This provides an automated, consistent and robust solution for determining reflectance signal within treated cells compared to control cells. The analysis determines the exact number of regions detected, providing major benefit for large scale experiments with ranges of conditions and treatments, crucial for the identification of specific factors involved in NP uptake. The software can also be extended to co-occurrence or colocalisation analysis. This can involve images from two modalities (as shown here with RCM and R-SIM) or can include endosomal segmentation, facilitating quantification of co-occurrence of NP within labelled cellular compartments (such as Pearson correlation values, M1 and M2 coefficients and object overlap). This facilitates a range of analyses of sample sizes of hundreds of cells within minutes indicating the time efficient nature of the technique, and highlighting the significant advantages of the proposed methods for large scale, high throughput NP-cell investigations.

This manuscript focuses on the acquisition and associated analyses of reflectance microscopy (RCM and R-SIM) applied to the investigation of SPIONs and cerium dioxide NP uptake into cancer cell models. This is primarily due to biomedical motivations in both diagnostics and cancer therapy. SPIONs and cerium dioxide offer potential candidates for drug delivery to cancer cells. SPIONs also hold great potential for contrast enhanced MRI, to enable early diagnosis and therefore enhance prognosis, hyperthermia treatment, and magnetic targeting and controlled release of therapeutic agents [9–11]. Despite the huge potential NPs offer, they sometimes failed to perform in the clinic [12]. The continued research into the behavior of NPs following cellular uptake will no doubt contribute to better drug design rational and lead to the production of successful therapies in the future. Cancer cell culture can therefore provide valuable insight into these behaviours in 2D monolayers, as shown here, and also in 3D tumour micro-environments using specialised dishes such as nano-imprinted culture plates (JSR micro) [62]. These multicellular tumour micro-environments can be investigated using reflectance microscopy to understand if and how NP uptake and translocation occurs in a 3D environment, and subsequently guide therapeutic NP design to hopefully facilitate efficacious results in clinical trials [12]. For example, the use of R-SIM in this case allowed us to identify precise correlation of NP with the lysosome, which could in turn facilitate nanodrug carrier designs that incorporate pH triggered drug release to allow therapeutic payloads to reach their target.

The strategies described have also been applied to lung cells, which can give insight into the effects following airborne exposure, important for NPs such as cerium dioxide that are released from vehicle exhausts. In this way, *in vitro*, models can give indications on the deleterious effect of NPs on cells originating from various organs. However, the issue of NP monitoring within whole organisms, not just *in vitro* cultures, is an area of high priority. A whole organism is significantly more complex than a single cell and therefore a host of parameters need to be assessed to systematically evaluate the health effects associated with NP exposure [63]. *In vivo* toxicity studies usually involve the inhalation, ingestion or injection of NP dose, followed by isolation of potential target organs and histopathological staining of resulting tissue sections [64]. Reflectance microscopy has been demonstrated for imaging of metallic quantum dots in *ex-vivo* epidermis and therefore may offer opportunities to investigate the specific target organ and associated effects of NP exposure in tissue sections [65].

Although the application documented here is directed towards nanosafety and nanomedecine, reflectance methods offer value for a broad range of investigations spanning multiple fields. RCM has been applied to the imaging of the focal contacts formed by cells attached to a surface, the morphology of red blood cells and the *in vivo* clinical diagnosis of a variety of pathologies, including skin, oral mucosa and cornea disease [31,32,66,67]. RCM has also been used to investigate the vesicle transport system in developing drosophila oocyte providing a real-time imaging method for innately reflective cellular constituents [68]. The availability of super-resolution reflectance (R-SIM) could provide valuable additions to investigations relating to these reflective samples with higher precision and detail. Furthermore, adopting a correlative workflow could potentially provide a means of combining dynamic live reflectance and fluorescence imaging data of processes such as disease pathogenesis, endocytosis, vesicle trafficking and ligand/receptor interactions with super-resolution imaging and localisation in reference space with TEM imaging.

## Materials and Methods

### Cell Culture and Maintenance

All cell culture techniques were performed under a sterile tissue culture hood (SterilGard, The Baker Company, Sanford, Maine, USA). All solutions and equipment were bought sterile or sterilised by autoclave when required.

Immortalized HeLa cervical carcinoma cells (ATCC) and A549 lung carcinoma cells (ATCC) were cultured at 37°C in a humidified CO_2_ incubator (Nuaire NU-5100 E/G Air Jacketed Automatic CO_2_ Incubator; Minnesota) at 5% CO_2_ in 10 cm petri dishes (Corning, New York, USA), containing Gibco Dulbecco’s Modified Eagle’s medium (DMEM) (Corning, New York, USA), supplemented with Foetal Bovine Serum (10% v/v) (FBS), penicillin (Corning, New York, USA), and streptomycin (100 μg/ml, Corning, New York, USA). This is subsequently referred to as Supplemented Culture Medium (SCM). Cells were grown to confluence and passaged using a standard trypsin-EDTA (0.25%: 0.2%) protocol (Invitrogen, UK).

### Nanoparticles

For the purpose of this proof of concept / workflow development, two representative NPs, known to show reflectance, were selected: SPIONs (Endomagnetics, Sysmex UK) and cerium dioxide synthesized and characterised as previously described. The core sizes are 4 nm and 8 nm respectively as measured by TEM (S1 Fig) [69]. Particles were sonicated for 15 minutes and subsequently dispersed in cell culture media containing 10% FBS at concentrations of 280 μg/ml (SPIONs) / 500 μg/ml (Cerium dioxide). Particles were applied to cells immediately upon preparation. Characterisation of the particle sizes in cell culture media has also been conducted to confirm that the particles were not agglomerated under the exposure conditions. Summary data on the particle characteristics is given in S1 Table.

### Cellular uptake of NPs

HeLa or A549 cells were cultured in 35mm gridded glass bottom dishes (MatTek Corporation, Maryland, USA) and incubated overnight in SCM (2 mL). At 50% confluence cells were treated with reflectant NPs, prepared as described, for 1 hour then incubated with CellTracker Orange (CTO) (Invitrogen, UK) diluted 1:1000 using the manufacturer’s standard protocol (Invitrogen, UK) for 30 minutes or Cell Tracker Deep Red (Invitrogen, UK) diluted using the standard protocol (Invitrogen, UK) for 30 minutes. LysoTracker was added to cells 30 minutes prior to the incubation end point following the standard protocol (Invitrogen, UK). Cells were then washed twice with PBS followed by SCM incubation for 30 min.

### Cellular fixation

Cells were washed 3 x 0.1 M Cacodylate Buffer (CB) and then fixed with 2% gluteraldehyde/ 4% paraformaldehyde in 0.1 M CB for 60 minutes, followed by 3 x 0.1 M CBB wash. MatTek dishes were then either left in CB for imaging, or mounted with Vectashield containing DAPI (Vector Laboratories Ltd, Peterborough, United Kingdom).

### Confocal Imaging

Images were taken on a Nikon A1R inverted confocal microscope (Nikon Corp, Japan). To set up the reflectance optical configuration in NIS elements the first dichroic mirror was set to B520/80 to allow light transmission, the fourth channel was set up for reflectance using the 488 nm laser, and all channel light paths were set to through. CTO was excited using the 561 nm laser and the 405 nm laser was used for DAPI imaging. Where present, lysosomal stain was excited using a 561 nm laser and cytoplasm stain (CTRD) replaced CTO, and was excited using the 640 nm laser. Grid squares were visualised and located using a 10X Plan apo λ 0.45 NA objective. Grid squares for imaging were picked based on certain criteria including central location within the grid and sparse cell density. Z-stacks from the coverslip to the top of the cell at a step size of 200 nm were acquired using a 60X apo 1.4 NA or a 100X apo 1.49 NA objective. Entire grid squares were imaged using large image capture (0.6mm squared) and stitching or single cells, picked based upon distinctive features and shapes for identification in subsequent imaging modalities, were imaged.

### Super-resolution reflectance Structured Illumination Microscopy (SIM) imaging

Following, or prior to, RCM imaging cells mounted in PBS or Vectashield were imaged with reflectance Structured Illumination Microscopy (SIM) (N-SIM, Nikon Corp., Japan) with an EM-CCD camera iXon3 DU-897E (Andor Technology Ltd.). To facilitate R-SIM a half mirror was placed in the light path by utilizing a filter cube that contains a half-mirror in place of the dichroic. The 488 nm laser was used to illuminate the sample using 2D-SIM imaging. CTO was excited using a 561 nm laser. Where present lysosomal stain was excited using a 561 nm laser and cytoplasm stain (CTRD) replaced CTO, and was excited using the 640 nm laser. First, the grid square was located on a 10X Plan apo λ 0.45 NA objective and the area of interest centred. The objective was switched to the 100X apo 1.49 NA and images were acquired either as entire grid squares (by use of the X-Y multipoint acquisition) or single cells were picked based on confocal location using the previously acquired images. Due to the high magnification and small field of view, acquisition of 0.5 mm^2^ in 15 focal planes using the N-SIM leads to acquisition, reconstruction and stitching times totalling over 24 hours; the final reconstruction provides a large amount of high resolution data, but also regions of blank reconstructed space where no cells are localised. Acquiring Z-stacks (200 nm Z-step) of individual cells provides a time efficient data acquisition, allowing reconstruction parameters to be tailored on a per cell basis, improving the finally reconstructed images. Z-stacks of the cells were therefore acquired to facilitate alignment with the correct plane in other modalities (RCM and TEM) with a step of 200 nm. Images were reconstructed using NIS elements SIM reconstruction software.

Acquiring RCM data first minimises photobleaching effects in the fluorescence channels that can occur during SIM acquisitions. Glass bottom Petri dishes with an etched alpha-numeric grid pattern allow successful cell relocation across modalities. A region of interest can be located using a low magnification objective lens (10X), followed by a high magnification acquisition of the specific region (RCM: 100X/100X. SIM: 100X). Z-stack acquisitions provide 3D information from RCM and R-SIM; however, the optical thickness of sections obtained will differ between modalities. The theoretical optical thickness in RCM is ~480 nm, whereas for SIM it is 685 nm. However, while RCM utilizes pinhole optics to exclude out of focus light from optical sections, SIM suffers from out of plane haze due to the widefield illumination path employed. Acquiring Z-stacks (200 nm Z-step) of individual cells provides a time efficient data acquisition, allowing reconstruction parameters to be tailored on a per cell basis, improving the finally reconstructed images.

### Processing for transmission electron microscopy (TEM)

Following light microscopy, cells were washed with 0.1 M PB buffer before a second fixation with 2.5% gluteraldehyde / 2% paraformaldehyde (EMS, Hatfield, Pennsylvania) in 0.1 M Cacodylate Buffer (CB) (1h –overnight) then washed with 3 x 0.1 M CB buffer. Cells were then stained with 2% osmium tetroxide (OsO_4_) (EMS, Hatfield, Pennsylvania) (1 hour) followed with 3 x dH_2_O washes. Cells were then fixed with uranyl acetate (1 hour) followed by washing with 3 x dH_2_O. Cells were then dehydrated with a series of ethanol washes x 2 (50%, 60%, 70%, 80%, 90% and 100%) before infiltration with 50:50 absolute alcohol:EmBed 812 (1 hour). EmBed 812 was made up as per the standard protocol for hard resin (EMS, Hatfield, Pennsylvania). Two subsequent infiltrations were performed (45 min) with EmBed 812 alone, before inverting and mounting on resin-filled embedding BEEM® capsules (EMS, Hatfield, Pennsylvania) with care taken to remove all bubbles from within the capsule. Samples were then baked overnight at 60°C in an oven (Thelco Laboratory Apparatus by Precision Scientific Co., India). Coverslips were separated from BEEM capsules by plunging into liquid nitrogen, and samples were then allowed to re-equilibrate with room temperature. The area of interest (0.5 mm^2^) was visualised, and trimmed and isolated with a sharp single edge razor blade (EMS, Hatfield, Pennsylvania) under a light microscope (Leica UltraCut UCT, Leica Microsystems Inc., IL, USA). Following trimming, 70 nm or 150 nm serial sections were cut using a Diatome diamond knife (EMS, Hatfield, Pennsylvania). Sections were then collected onto 200 mesh copper (Cu) grids or slotted grids (EMS, Hatfield, Pennsylvania) that had been pre-treated with alcohol. Samples were then stored for staining.

Grids containing sections were stained by inverting on top of small blobs of 2% uranyl acetate (10 minutes) (EMS, Hatfield, Pennsylvania) inside petri dishes; grids were then washed with dH_2_O and air dried before repeating this step with Reynolds lead citrate (CaCO_3_ crystals used to remove air from within the chamber). Following this, grids were washed with dH_2_O and left to dry before TEM imaging.

### TEM imaging

TEM imaging was carried out on an FEI Tecnai G2 Spirit at 80 KeV (FEI, Center for Advanced Microscopy, Northwestern University, Chicago, IL). Images were taking with a Gatan imaging camera. TEM grids were loaded and cells of interest from RCM and/or SIM experiments were located on low (690 x) magnification, and then imaged at higher magnification (up to 49,000 x) to visualise individual NPs, and clusters of NPs.

### Image analysis

MATLAB R2011b was used in the analysis of both RCM and R-SIM images. Maximum intensity Z- projections were created with MATLAB using Z-stack multi-plane acquisitions. RCM and R-SIM overlays were made using MATLAB using the point selection tool ‘cpselect’ and the ‘imregister’ function. Pixel size was equalised and rigid body realignments allowed the channels to be combined as overlays. Cell segmentation was achieved using Gaussian smoothing followed by K-means clustering (nClusters = 2) of CTO cytoplasmic stain. NP segmentation was achieved by forming a 3-dimensional matrix of images including treated and control images, normalised together, and then segmented using K-means clustering and intensity thresholding. Quantifications were then performed in loops for automation, using a binary overlay of the cell onto the NP channel, and parameters were assessed such as connected component number and connected component area. This information was then visualised graphically using MATLAB. For the comparison of detected regions, binary images created from SIM and RCM NP segmentations were analysed. The binary image gave the total ‘number of connected components’; using the bwconncomp function in each image. The images were then multiplied together and the number of connected components that appear in both images was computed, and subsequently the percentage of the total detected (Number in multiplied image divided by the number in initial binary image) that correlate was calculated and presented graphically.

### Image registration

Co-alignments of TEM and reflectance images were performed in MATLAB. For the reflectance image coalignment (SIM and RCM max intensity projections), between 3 and 6 points pairs, were selected on images that correspond to one another. Following user selection of points, MATLAB calculates the transformation matrix that best aligns the pairs, using ‘rigid’ registration. Alternatively, MATLAB has a built in function called ‘imregister’. This can be initialized using the ‘multimodal’ configuration setup. ‘Imregister’ can successfully automate the alignment of RCM and R-SIM images using an intensity based method, however, in order to be successful images must first be smoothed and background subtracted (to negate the contribution of background reflectance to the intensity registration). Overlays were created using MATLAB, however pseudocolour was created in ImageJ. Coalignment of adjacent TEM slices was achieved using an intensity based registration function (imregister) as a ‘rigid’ transformation with parameters that facilitated alignment. Coalignment of reflectance and TEM images was achieved using the cpselect function with between 3 and 10 matching points. The transformation matrix was then calculated and applied as an ‘affine’ transformation, to cope with cell shrinkage. The CPD algorithm was explored for the fully automated realignment of matching slices based on nuclear segmentation from light and electron microscopy.

### Statistical analyses

Statistical significance was determined by Students T-tests. * P<0.05, ** P = <0.01, *** P<0.001.

## Supporting Information

S1 Table Properties of nanoparticles used in this study. S1 Table displays information regarding the size of the NPs used (SPIONs and cerium dioxide). Core size is the size of the metallic NP core. Nominal size is the size that is given by the manufacturers and Size in SCM is the size measured in DLS (NP + protein corona). Zeta potential is the charge in DI water.
(PNG)

S1 Fig TEM micrographs of SPIONs and cerium dioxide NPs deposited onto grids. TEM micrographs of A) SPIONs (Sigma-Aldrich) B) SPIONs (Sienna^+^, Endomagnetics) and C) cerium dioxide NPs [69].
(TIF)

S2 Fig Semi-quantitative analysis of NP uptake. Result of automated analysis of NP uptake in cells. Quantification of NP uptake is displayed as the number of connected components. A connected component refers to a cluster of NPs detected as intensity in the reflected light image. Uptake is seen using RCM of cells exposed to SPIONs (69 cells) and cerium dioxide (68 cells) NPs, and not in the control cells (58 cells). SIM analysis of cells exposed to SPIONs (12 cells) and cerium dioxide (12 cells) NPs also demonstrate uptake with no uptake seen in the controls cells (10 cells). Results are a combination of 3 or more experiments carried out on separate days. The mean number of connected components and STD is plotted; students T-Test gave P value <0.001.
(TIF)

S3 Fig Comparison of data obtained from RCM using a 60X objective and SIM reflectance. Maximum intensity Z-projection images of a HeLa cell treated with cerium dioxide NPs, acquired with RCM (60X 1.40 NA) and SIM reflectance (100X 1.49 NA). RCM imaging volume is 8.4 μm and SIM 6.8 μm. Images show CTO (red) cytoplasmic stain, DAPI (blue) nuclear stain and NP signal (grey). Overlay of the cerium dioxide NP regions shows particles detected on RCM (blue) and SIM (grey). White boxes display a sample of regions where RCM detects one spot and SIM detects multiple spots, illustrating the enhanced resolution of SIM.
(TIF)

S4 Fig Live cell reflectance confocal time lapse stills indicating the colocalisation of cerium dioxide NPs with lysosomes in A549 cells. Images show reflectant NPs (grey), DAPI nuclear stain (blue) and LysoTracker Red stain (red). Time lapse videos were taken to visualise NP uptake and trafficking into vesicles over the course of 15/30 minutes. Stills from 1, 10 and 19 minutes are shown. NPs are evident and in some cases can be seen to colocalise with red lysosomal stain (white arrows).
(TIF)

S5 Fig TEM images showing the individual sections that comprise the composite shown in Fig 7. Sections are 150nm thick and can be combined to create a minimum intensity projection of 300 nm thick to better represent the thickness of RCM. A section at 1900X magnification is also used to allow visualisation of the entire cell, with increased magnification at regions of NP uptake in the centre of the cell.
(TIF)

S6 Fig TEM images showing regions of electrodensity in HeLa cell treated with cerium dioxide NPs. Images showing NPs within HeLa cells (A) and regions where no NPs are detected but electron density appears to be observed at low magnification (B and C).
(TIF)

S7 Fig Cellular uptake and localisation of SPIONs (Sigma-Aldrich) visualized by RCM and TEM. CREM (RCM and TEM) using fixed A549 cells treated with SPIONs. The cell outlines in both TEM and RCM are highly preserved facilitating identification of the same cell. The ultastructure of the cell is preserved and the sub-cellular vesicular localisation of NPs is evident. Individual NPs can be visualised at high magnification of 30000X with TEM. Reflectance overlay is one optical section of a confocal stack, with optical thickness being approximately the FWHM_axial_ calculated to be 954 nm. Black arrows indicate regions where NP localisation to vesicles can be observed.
(TIF)

S8 Fig FWHM and Z-PSF of intracellular cerium dioxide NPs. Z-Y and Z views of R-SIM Z-stacks (A) and RCM (B) showing the Z-PSF of the same NP signal using each technique. R-SIM has a narrower Z-PSF despite the larger optical slice thickness. Identification of multiple regions on images from both RCM and R-SIM indicated the effect this has on particle inclusion across imaged Z-planes. Intensity line scans were plotted along the Z axis at these regions and the FWHM measured. The graph (C) represents 20 regions with the STD plotted. Students T-Test gave P-Value of 5.2x12^-12^ indicating the groups are significantly different to one another. The FWHM of line intensity scans can be used to measure Z-PSF, examples of the line intensity plot on R-SIM (D) and RCM (E) are shown.
(TIF)

S9 Fig Automated affine transformation for image realignment using coherent point drift. Example of automated affine transformation accurately registering images from different modalities (TEM and RCM (A) and TEM and SIM (B)). Segmentation of DAPI nuclear regions from TEM and FCM facilitate fully automated alignment using CPD algorithm. This is currently restricted to specific images with well-defined features to register to, such as a double nuclei [60].
(TIF)

S1 Video Live cell reflectance confocal time lapse indicating the colocalisation of cerium dioxide NPs with lysosomes in A549 cells. Images show reflectant NPs (grey), DAPI nuclear stain (blue) and LysoTracker Red stain (red). Time lapse images were taken in 1 minute increments to visualise NP uptake and trafficking into lysosomes over the course of 20 minutes. S4 Fig shows stills from this video.
(AVI)

S1 Box Summary of experimental conclusions for RCM. (PNG)

S2 Box Summary of experimental conclusions for R-SIM. (PNG)

## Abbreviations

CB: Cacodylate Buffer
CLSM: Confocal Laser Scanning Microscopy
CPD: Coherent Point Drift
CREM: Correlative Reflectance Electron Microscopy
CTO: CellTracker Orange
DAPI: 4’,6-diamdino-2phenylindeo
EM: Electron Microscopy
FFT: Fast Fourier Transform
FWHM: Full Width at Half Maximum
IRM: Interference Reflectance Microscopy
ICP: Iterative Closest Point
NP(s): Nanoparticle(s)
NA: Numerical Aperture
PB: Phosphate Buffer
PBS: Phosphate Buffered Saline
RCM: Reflectance Confocal Microscopy
RI: Refractive Index
SCM: Supplemented Culture Media
SEM: Standard Error of Mean
STD: Standard Deviation
SIM: Structured Illumination Microscopy
SPIONs: Super Paramagnetic Iron Oxide Nanoparticles
SNR: Signal to Noise Ratio
TEM: Transmission Electron Microscopy
TIRM: Total Internal Reflectance Microscopy
