# Supplementary figures and images for: Comparison of Confocal and Super-Resolution Reflectance Imaging of Metal Oxide Nanoparticles
Source: PLoS One. 2016 Oct 3;11(10):e0159980. doi: 10.1371/journal.pone.0159980 (PMC5047631; doi:10.1371/journal.pone.0159980)

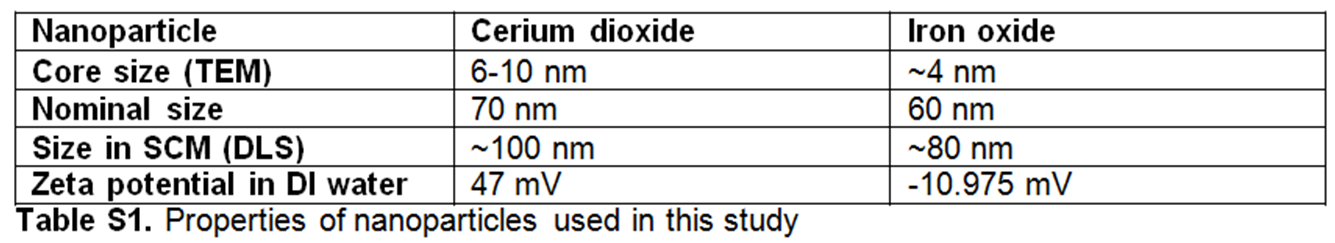

Supplement: S1 Table — displays information regarding the size of the NPs used (SPIONs and cerium dioxide). Core size is the size of the metallic NP core. Nominal size is the size that is given by the manufacturers and Size in SCM is the size measured in DLS (NP + protein corona). Zeta potential is the charge in DI water. (PNG) [file pone.0159980.s001.png]

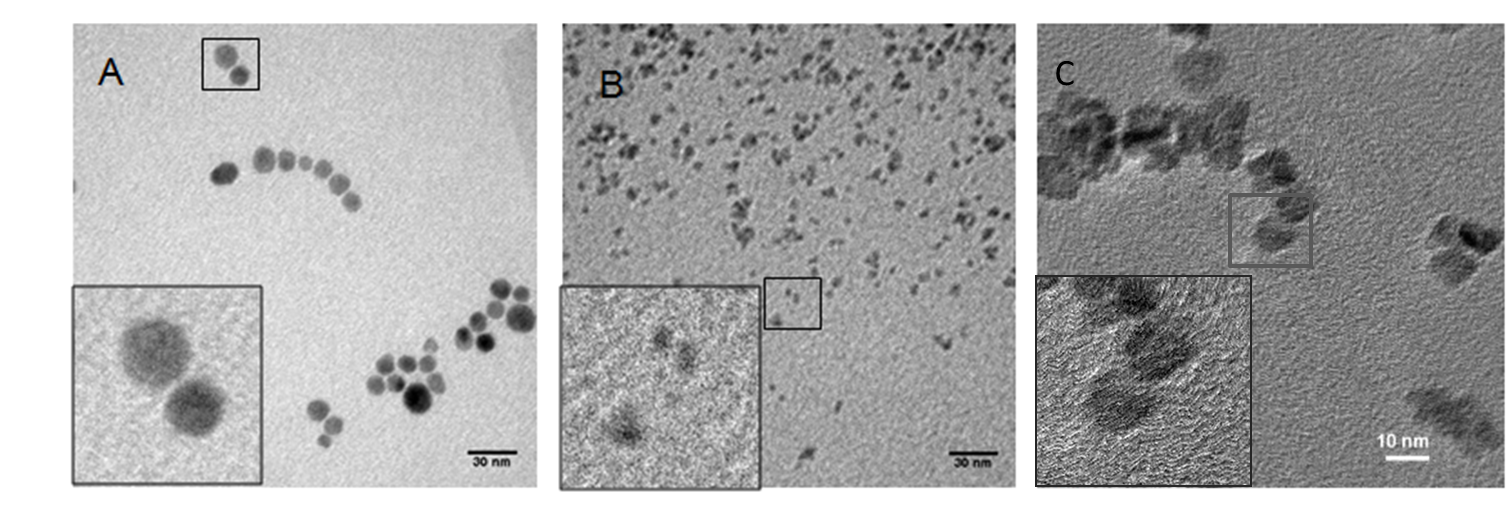

Supplement: S1 Fig — TEM micrographs of A) SPIONs (Sigma-Aldrich) B) SPIONs (Sienna+, Endomagnetics) and C) cerium dioxide NPs [69]. (TIF) [file pone.0159980.s002.tif]

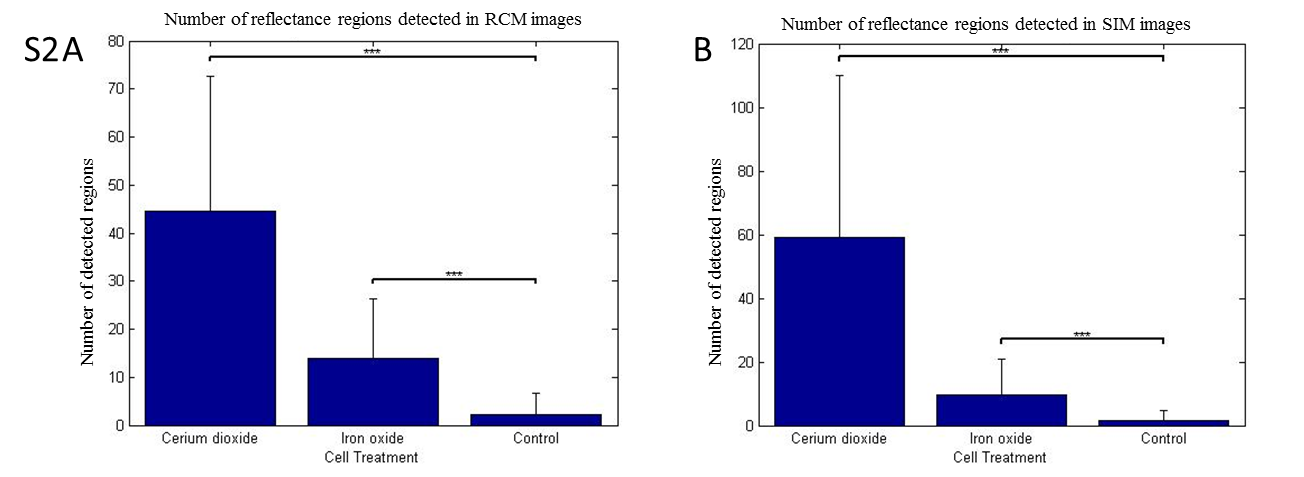

Supplement: S2 Fig — Result of automated analysis of NP uptake in cells. Quantification of NP uptake is displayed as the number of connected components. A connected component refers to a cluster of NPs detected as intensity in the reflected light image. Uptake is seen using RCM of cells exposed to SPIONs (69 cells) and cerium dioxide (68 cells) NPs, and not in the control cells (58 cells). SIM analysis of cells exposed to SPIONs (12 cells) and cerium dioxide (12 cells) NPs also demonstrate uptake with no uptake seen in the controls cells (10 cells). Results are a combination of 3 or more experiments carried out on separate days. The mean number of connected components and STD is plotted; students T-Test gave P value <0.001. (TIF) [file pone.0159980.s003.tif]

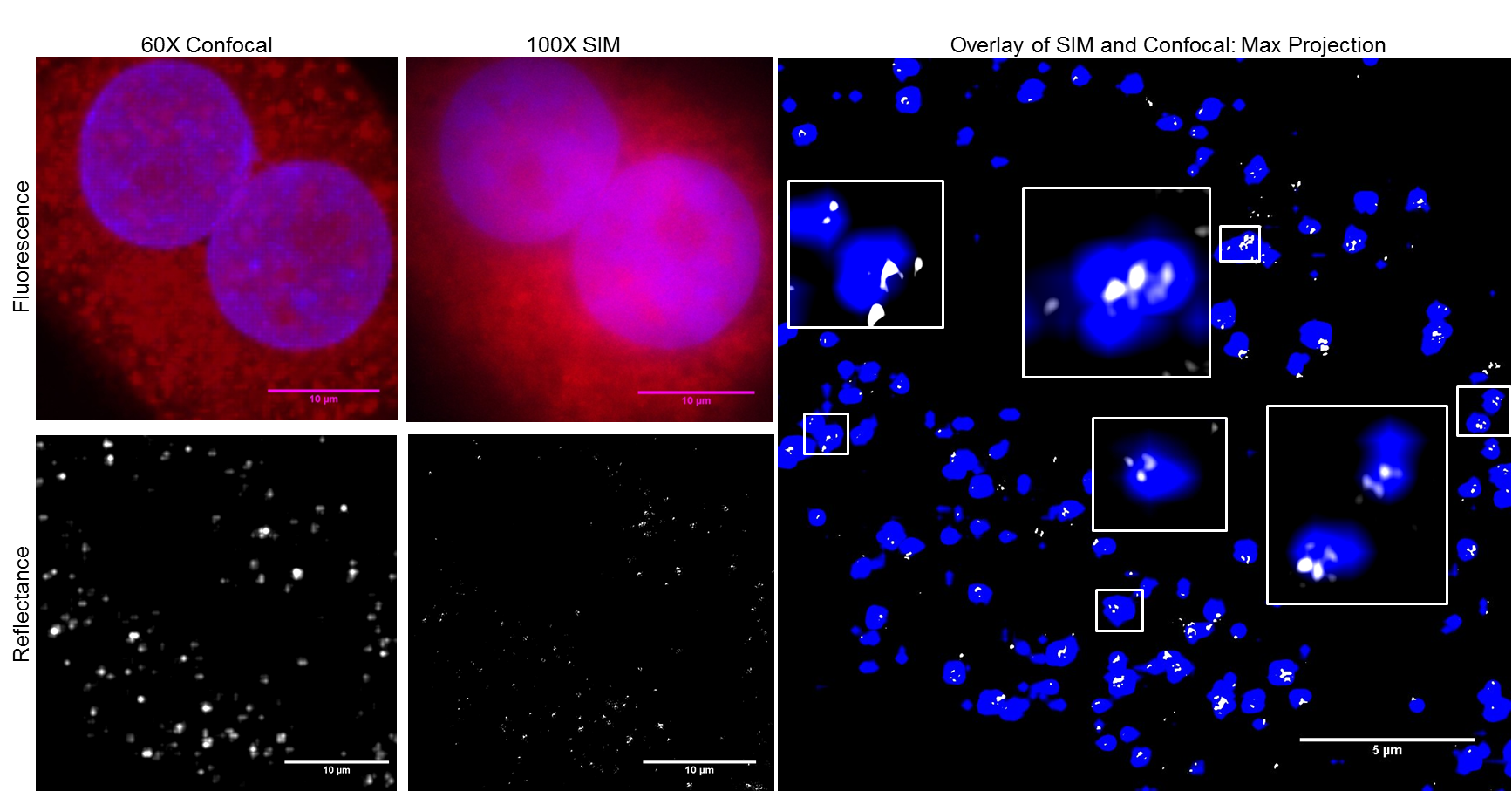

Supplement: S3 Fig — Maximum intensity Z-projection images of a HeLa cell treated with cerium dioxide NPs, acquired with RCM (60X 1.40 NA) and SIM reflectance (100X 1.49 NA). RCM imaging volume is 8.4 μm and SIM 6.8 μm. Images show CTO (red) cytoplasmic stain, DAPI (blue) nuclear stain and NP signal (grey). Overlay of the cerium dioxide NP regions shows particles detected on RCM (blue) and SIM (grey). White boxes display a sample of regions where RCM detects one spot and SIM detects multiple spots, illustrating the enhanced resolution of SIM. (TIF) [file pone.0159980.s004.tif]

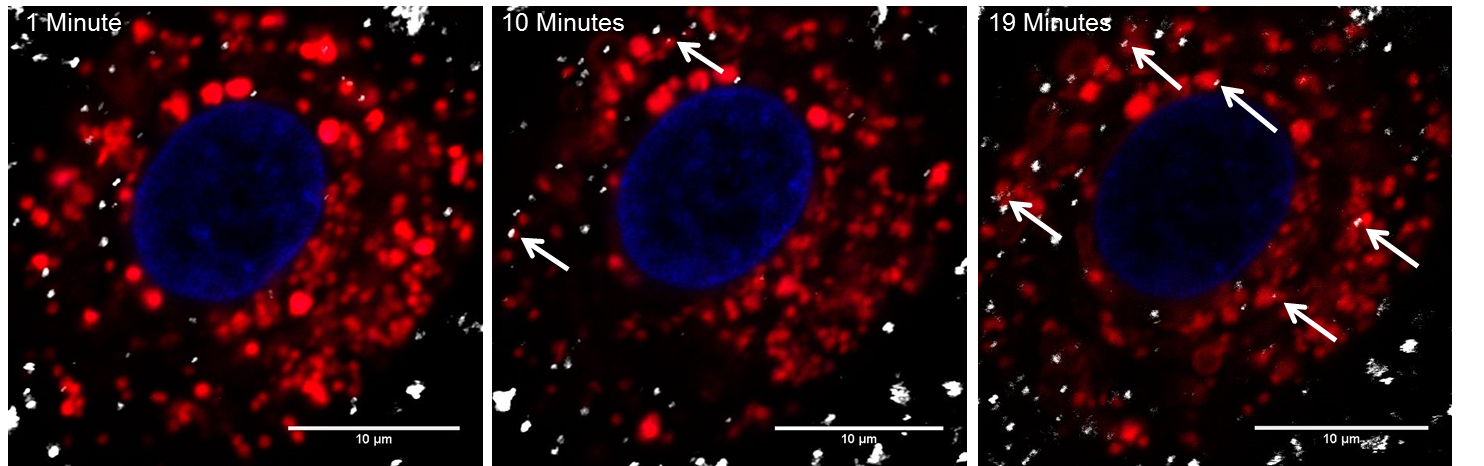

Supplement: S4 Fig — Images show reflectant NPs (grey), DAPI nuclear stain (blue) and LysoTracker Red stain (red). Time lapse videos were taken to visualise NP uptake and trafficking into vesicles over the course of 15/30 minutes. Stills from 1, 10 and 19 minutes are shown. NPs are evident and in some cases can be seen to colocalise with red lysosomal stain (white arrows). (TIF) [file pone.0159980.s005.tif]

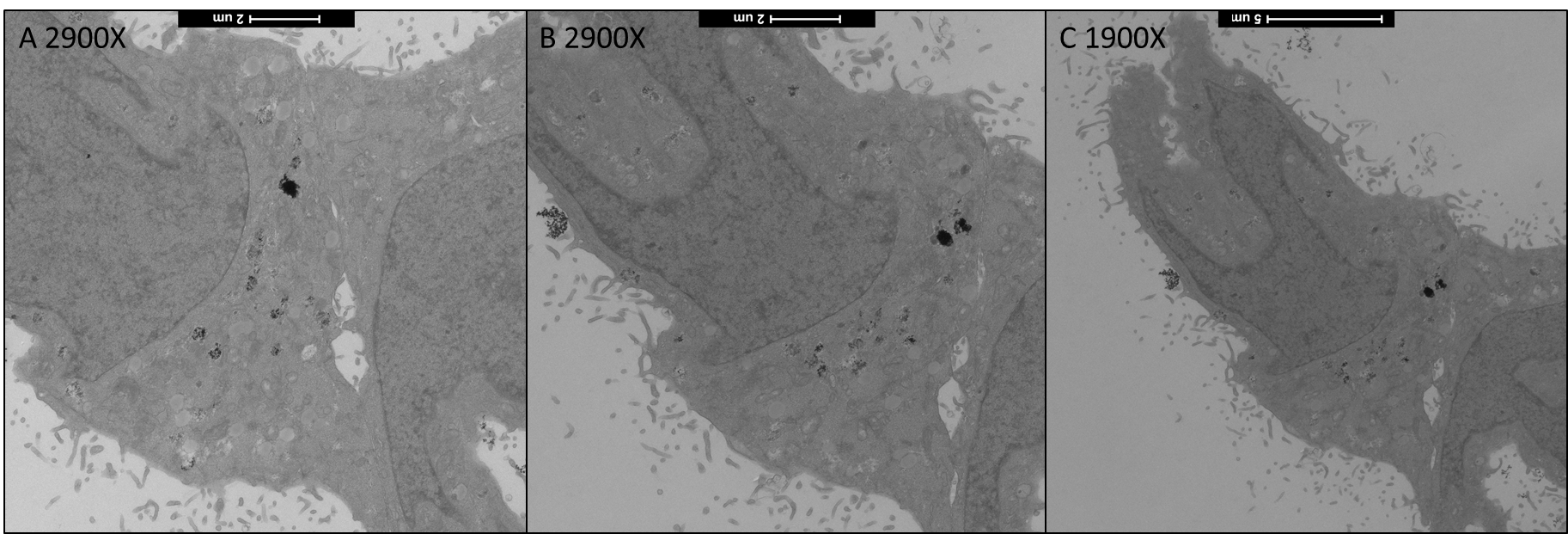

Supplement: S5 Fig — Sections are 150nm thick and can be combined to create a minimum intensity projection of 300 nm thick to better represent the thickness of RCM. A section at 1900X magnification is also used to allow visualisation of the entire cell, with increased magnification at regions of NP uptake in the centre of the cell. (TIF) [file pone.0159980.s006.tif]

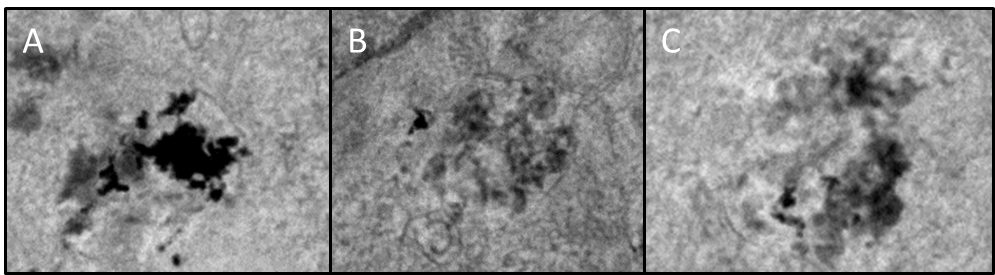

Supplement: S6 Fig — Images showing NPs within HeLa cells (A) and regions where no NPs are detected but electron density appears to be observed at low magnification (B and C). (TIF) [file pone.0159980.s007.tif]

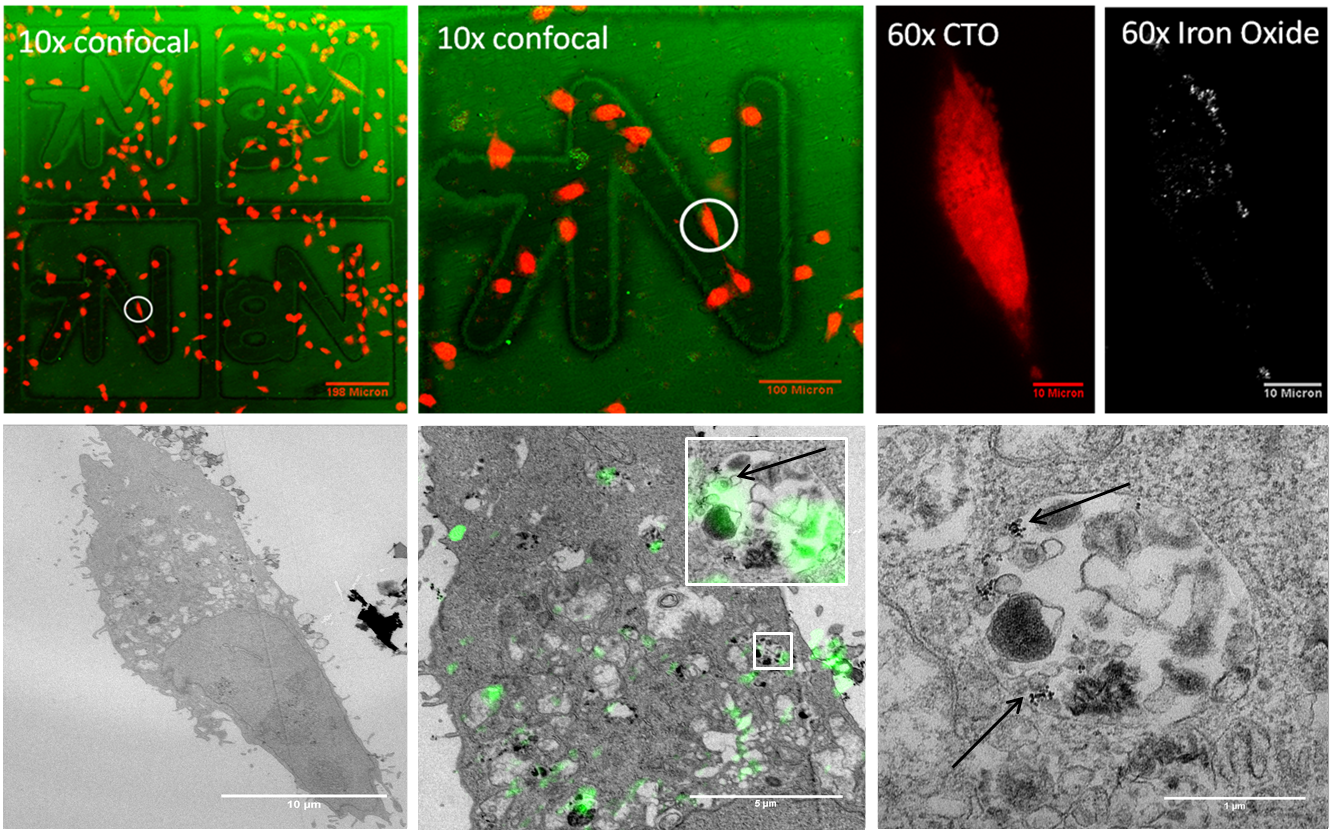

Supplement: S7 Fig — CREM (RCM and TEM) using fixed A549 cells treated with SPIONs. The cell outlines in both TEM and RCM are highly preserved facilitating identification of the same cell. The ultastructure of the cell is preserved and the sub-cellular vesicular localisation of NPs is evident. Individual NPs can be visualised at high magnification of 30000X with TEM. Reflectance overlay is one optical section of a confocal stack, with optical thickness being approximately the FWHMaxial calculated to be 954 nm. Black arrows indicate regions where NP localisation to vesicles can be observed. (TIF) [file pone.0159980.s008.tif]

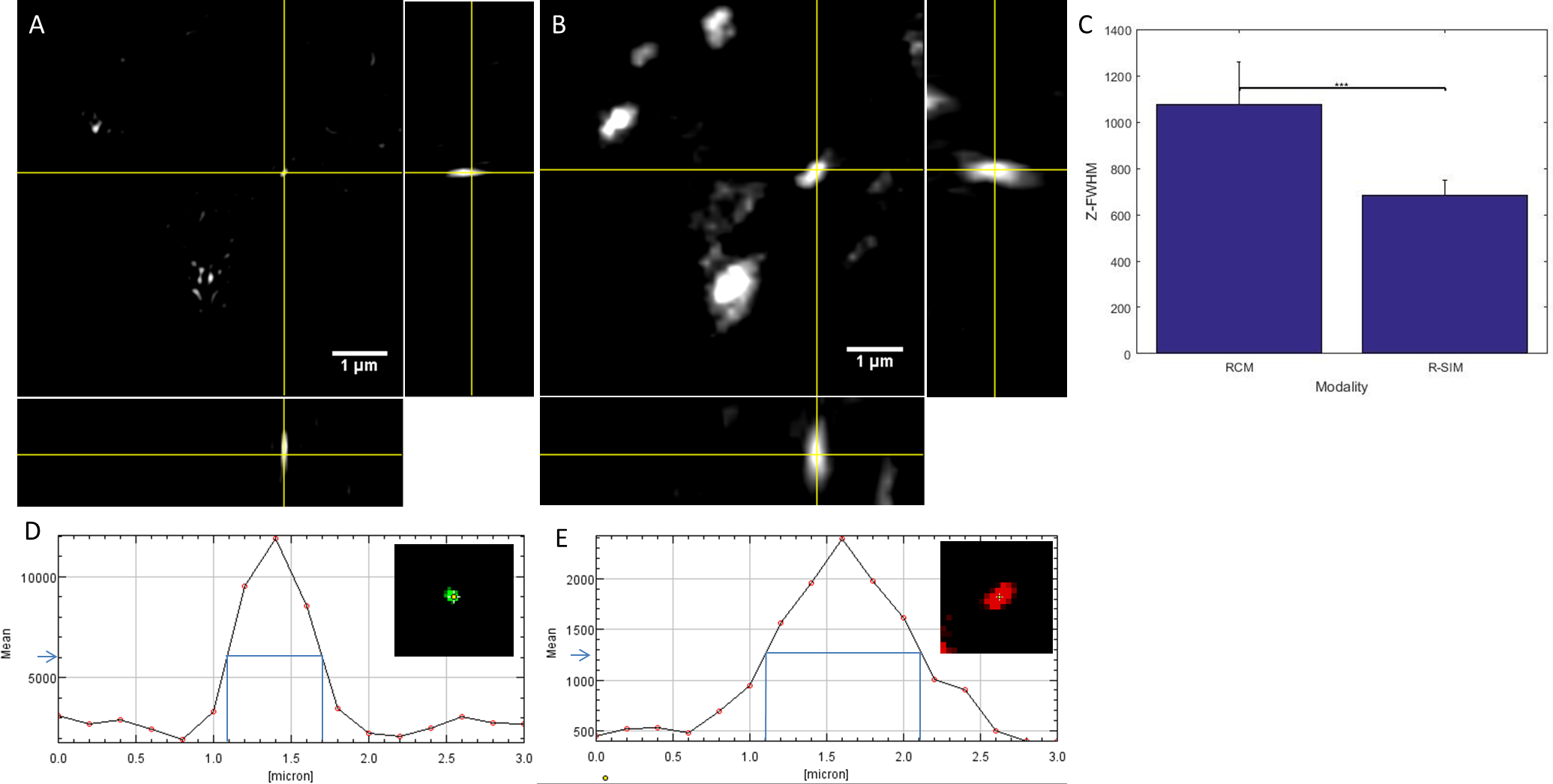

Supplement: S8 Fig — Z-Y and Z views of R-SIM Z-stacks (A) and RCM (B) showing the Z-PSF of the same NP signal using each technique. R-SIM has a narrower Z-PSF despite the larger optical slice thickness. Identification of multiple regions on images from both RCM and R-SIM indicated the effect this has on particle inclusion across imaged Z-planes. Intensity line scans were plotted along the Z axis at these regions and the FWHM measured. The graph (C) represents 20 regions with the STD plotted. Students T-Test gave P-Value of 5.2x12-12 indicating the groups are significantly different to one another. The FWHM of line intensity scans can be used to measure Z-PSF, examples of the line intensity plot on R-SIM (D) and RCM (E) are shown. (TIF) [file pone.0159980.s009.tif]

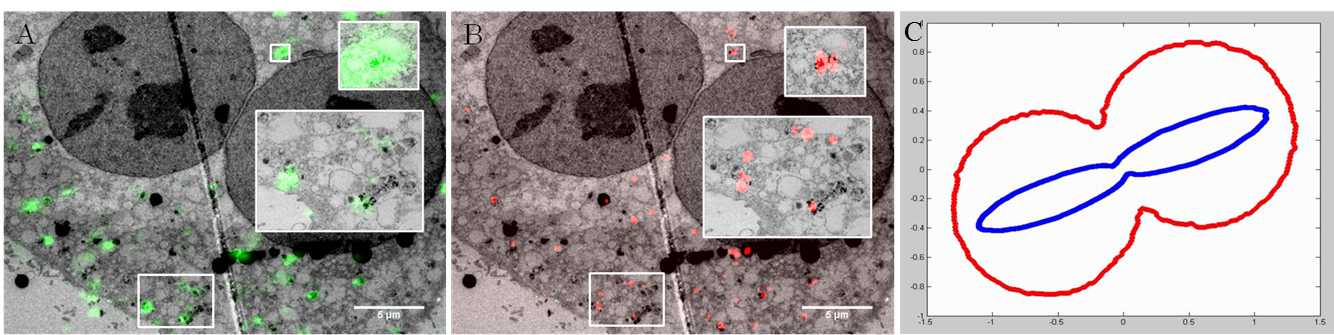

Supplement: S9 Fig — Example of automated affine transformation accurately registering images from different modalities (TEM and RCM (A) and TEM and SIM (B)). Segmentation of DAPI nuclear regions from TEM and FCM facilitate fully automated alignment using CPD algorithm. This is currently restricted to specific images with well-defined features to register to, such as a double nuclei [60]. (TIF) [file pone.0159980.s010.tif]

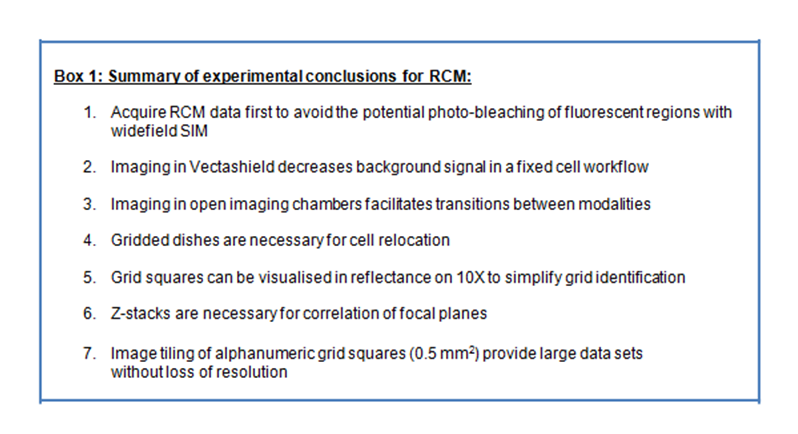

Supplement: S1 Box — (PNG) [file pone.0159980.s012.png]

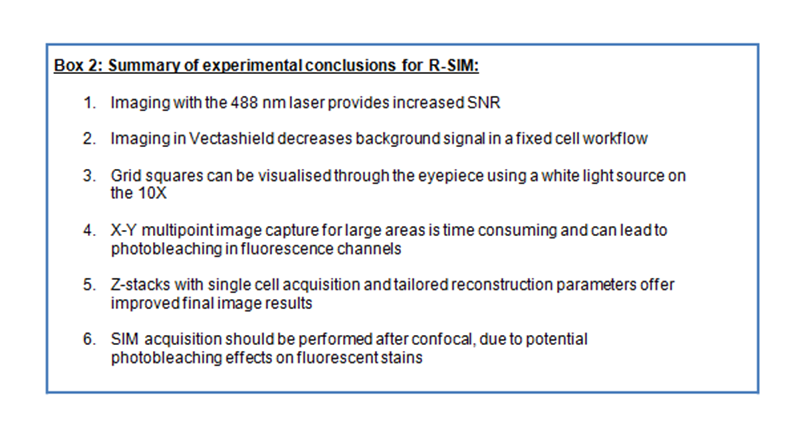

Supplement: S2 Box — (PNG) [file pone.0159980.s013.png]
